# Understanding adefovir pharmacokinetics as a component of a transporter phenotyping cocktail

**DOI:** 10.1007/s00228-024-03673-x

**Published:** 2024-03-28

**Authors:** Qian Dong, Chunli Chen, Max Taubert, Muhammad Bilal, Martina Kinzig, Fritz Sörgel, Oliver Scherf-Clavel, Uwe Fuhr, Charalambos Dokos

**Affiliations:** 1 Department I of Pharmacology, Center for Pharmacology, Faculty of Medicine and University Hospital Cologne, University of Cologne, Gleueler Straße 24, Cologne, 50931 Germany; 2 Heilongjiang Key Laboratory for Animal Disease Control and Pharmaceutical Development, College of Veterinary Medicine, Northeast Agricultural University, 600 Changjiang Road, Xiangfang District, Harbin, 150030 People’s Republic of China; 3 Department of Clinical Pharmacy, Institute of Pharmacy, University of Bonn, Bonn, Germany; 4 Institute for Biomedical and Pharmaceutical Research, Nürnberg-Heroldsberg, Germany; 5 Department Pharmazie, Ludwig-Maximilians-Universität München, Butenandtstr. 5, 81377 München, Germany

**Keywords:** Adefovir, Population pharmacokinetics, Nonlinear renal elimination, OAT1-mediated drug-drug interactions

## Abstract

**Purpose:**

Adefovir (as dipivoxil) was selected as a probe drug in a previous transporter cocktail phenotyping study to assess renal organic anion transporter 1 (OAT1), with renal clearance (CL_R_) as the primary parameter describing renal elimination. An approximately 20% higher systemic exposure of adefovir was observed when combined with other cocktail components (metformin, sitagliptin, pitavastatin, and digoxin) compared to sole administration. The present evaluation applied a population pharmacokinetic (popPK) modeling approach to describe adefovir pharmacokinetics as a cocktail component in more detail.

**Methods:**

Data from 24 healthy subjects were reanalyzed. After establishing a base model, covariate effects, including the impact of co-administered drugs, were assessed using forward inclusion then backward elimination.

**Results:**

A one-compartment model with first-order absorption (including lag time) and a combination of nonlinear renal and linear nonrenal elimination best described the data. A significantly higher apparent bioavailability (73.6% vs. 59.0%) and a lower apparent absorption rate constant (2.29 h^−1^ vs. 5.18 h^−1^) were identified in the combined period compared to the sole administration period, while no difference was seen in renal elimination. The population estimate for the Michaelis-Menten constant (K_m_) of the nonlinear renal elimination was 170 nmol/L, exceeding the observed range of adefovir plasma maximum concentration, while the maximum rate (V_max_) of nonlinear renal elimination was 2.40 µmol/h at the median absolute estimated glomerular filtration rate of 105 mL/min.

**Conclusion:**

The popPK modeling approach indicated that the co-administration primarily affected the apparent absorption and/or prodrug conversion of adefovir dipivoxil, resulting in the minor drug-drug interaction observed for adefovir as a victim. However, renal elimination remained unaffected. The high K_m_ value suggests that assessing renal OAT1 activity by CL_R_ has no relevant misspecification error with the cocktail doses used.

**Supplementary Information:**

The online version contains supplementary material available at 10.1007/s00228-024-03673-x.

## Introduction

Membrane transporter proteins play a key role in the pharmacokinetics of drugs and are a potential target for transporter-based drug-drug interactions (DDIs) [1]. The “cocktail approach,” which uses a combined administration of selective probe drugs for various transporters, is a valuable approach for the simultaneous investigation of several DDIs in a single clinical trial [2]. Although several cocktails have been established [3–5], the specificity of probe substances and the suitability of their pharmacokinetic (PK) parameters for characterizing transporter activities need to be investigated in more detail.

The organic anion transporter 1 (OAT1; gene name SLC22A6), predominantly located in the basolateral membrane of renal proximal tubular cells and mainly responsible for renal disposition of numerous prescribed drugs (e.g., diuretics and antivirals) [6], has been highlighted by regulatory agencies as a key transporter involved in potential DDIs [7, 8]. In order to establish a “more selective” cocktail, according to the suggestions by the FDA guideline [7], adefovir dipivoxil was selected as a specific probe drug for renal OAT1 activity in a previous clinical DDI cocktail study [3], as it is highly selective for OAT1 in vitro [9]. In addition, renal clearance (CL_R_), which only depends on renal excretion driven by plasma concentrations and is independent of other PK processes (e.g., drug absorption or conversion rate form its prodrug), was selected as the appropriate and practical primary metric to reflect renal OAT1 activity [3]. While the use of renal secretion of adefovir to assess OAT1 activity is considered the gold standard, this metric depends on a precise measurement of glomerular filtration rate (GFR) and fraction unbound (fu), which were unavailable in the study [3]. In the case of adefovir with fu close to 1 [10], the unavailability of this parameter however is of little relevance.

Although the primary metric supported the absence of relevant interactions of adefovir with the other components of the cocktail (including 100 mg sitagliptin, 500 mg metformin, 2 mg pitavastatin, and 0.5 mg digoxin), an approximately 20% increase in systemic exposure for adefovir was observed during concomitant administration [3]. This indicated that there might still be some minor inhibition of OAT1, and/or an effect on other PK processes of adefovir might be present [3]. Extended in vitro analyses, however, did not support an effect of the other cocktail components on OAT1 activity [11]. Unfortunately, the non-compartmental analysis (NCA) applied in this trial could not explain the changes of PK processes in more detail.

Understanding the minor DDI observed for the victim drug adefovir in more detail also needs to take into account that OAT1-mediated secretion of adefovir (almost 60% of renal elimination [2]) might be saturable at studied concentrations. While this hypothesis might not be fully supported by in vitro findings, since adefovir peak plasma concentrations (C_max_) obtained in the clinical trial (5.56–91.0 nmol/L following a single dose of 10 mg adefovir dipivoxil [3]) were well below the Michaelis-Menten constant (K_m_, mean ± standard deviation [SD] 23.8 ± 4.2 µmol/L) observed in the studies [12, 13], there is evidence that such saturation may exist in vivo. Nonlinearities in adefovir C_max_ and/or area under the curve (AUC) have been reported in infants, children infected with HIV, and in adult volunteers [14, 15]. However, a detailed description of the PK processes contributing to the renal elimination of adefovir is currently lacking. If such non-linearities are not properly considered, the reliability of adefovir metrics for detection and quantification of potential DDIs might be affected in the cocktail.

Given these challenges, population pharmacokinetic (popPK) modeling might allow to gain a more mechanistic insight into the potential PK processes involved in the minor DDI observed for adefovir and to identify a possible nonlinear elimination process more precisely.

## Materials and methods

### Clinical study

Data from 24 healthy subjects in the previous clinical trial were reanalyzed [3]. The trial (ClinicalTrials.gov identifier: NCT02743260) was approved by the Ethics Committee of the Medical Faculty of the University of Cologne, Germany (application number 15-421; approval date: February 19, 2016). Conductance adhered to the pertinent version of the Declaration of Helsinki and to International Conference on Harmonization guidelines for Good Clinical Practice. All subjects gave informed consent. In this trial, a single 10 mg dose of adefovir dipivoxil was given alone and in combination with four aforementioned probe drugs in the reference and test periods, respectively. Plasma and urine concentrations of adefovir were measured up to 24 h using a validated HPLC–MS/MS method [16]. Lower limits of quantification (LLOQs) for adefovir were 0.998 nmol/L in plasma and 0.382 µmol/L in urine, respectively [16]. The PK parameters of adefovir in reference and test periods were initially estimated via NCA using Phoenix WinNonlin™ (Version 7.0, Certara, NJ, USA), based on the concentration profiles in plasma and urine [3].

### Basic population pharmacokinetic analysis

A popPK model of adefovir was developed using the nonlinear mixed-effects modeling program NONMEM 7.5.0 (ICON plc, Dublin, Ireland), Perl speaks NONMEM (PsN 5.2.6), and Pirana 3.0.0 (Certara, Princeton, NJ). R version 4.2.1 (R Foundation for Statistical Computing, Vienna, Austria) was used to build figures for model evaluations and for statistical summaries. The base model was developed starting from a one-compartment model, and model complexity was increased step by step. Inter-individual (IIV) and inter-occasion variability (IOV) of PK parameters were estimated assuming log-normally distributed individual PK parameters, and additive, proportional, and combined residual error models were evaluated. To identify a suitable base model, we focus on Bayesian information criterion (BIC)-based comparisons, which penalize model complexity and mitigate the risk of overfitting [17].

The elimination of adefovir was modeled with two components: renal clearance (CL_R_) and nonrenal clearance (CL_NR_). Since adefovir exhibits negligible plasma protein binding (< 3.0%) [10] and was not quantified in the study, we assumed that fu = 1 for modeling purposes. Employing a one-compartment model with first-order absorption, two elimination models were considered. “Model 1” assumed linear kinetics for both renal and nonrenal elimination. In “Model 2”, renal elimination followed Michaelis-Menten-type nonlinear kinetics, while nonrenal elimination retained linear kinetics. The details of these models are presented in Table 1.

**Table 1:** Summary of population pharmacokinetic models

Model		Model 1	Model 2
Absorption model		First-order absorption with lag time	
Elimination model	CL_R_	Linear	Michaelis-Menten-type nonlinear
	CL_NR_	Linear	Linear
Differential equation		$$\begin{array}{c}\frac{{dA}_{1}}{dt}=-{K}_{a}{A}_{1}\end{array}$$ $$\begin{array}{c}\frac{{dA}_{2}}{dt}={K}_{a}{A}_{1}-{K}_{NR}{A}_{2}-{K}_{R}{A}_{2}\end{array}$$ $$\begin{array}{c}\frac{{dA}_{3}}{dt}={K}_{R}{A}_{2}\end{array}$$	$$\begin{array}{c}\frac{{dA}_{1}}{dt}=-{K}_{a}{A}_{1}\end{array}$$ $$\begin{array}{c}\frac{{dA}_{2}}{dt}={K}_{a}{A}_{1}-{K}_{NR}{A}_{2}-\frac{{V}_{max}{A}_{2}/V}{{K}_{m}+{A}_{2}/V}\end{array}$$ $$\begin{array}{c}\frac{{dA}_{3}}{dt}=\frac{{V}_{max}{A}_{2}/V}{{K}_{m}+{A}_{2}/V}\end{array}$$

*CL*_*R*_ renal clearance, *CL*_*NR*_ nonrenal clearance, *t* time, *A* adefovir amount in a compartment, adefovir amounts in the absorption, central, and urine compartments are represented by A_1_, A_2_, and A_3_, respectively, *K*_*a*_ first-order absorption rate constant, *V* volume of distribution in the central compartment, *K*_*NR*_ linear nonrenal elimination rate constant, *K*_*R*_ linear renal elimination rate constant, *K*_*m*_ Michaelis-Menten constant for nonlinear renal elimination, *V*_*max*_ maximum rate of nonlinear renal elimination

### Covariate evaluation

For covariate evaluations, we utilized objective function value (OFV)-based statistical tests, employing a forward inclusion and backward elimination approach to investigate the influence of co-administered drugs, demographic, and physiological variables. We evaluated the impact of concurrent administration of cocktail components on adefovir PK by separately determining all parameters for both reference and test periods after establishing a reasonable base model. To identify the impact of demographic and physiological factors on PK parameters, we individually added variables like age, sex, body weight, body mass index (BMI), body surface area (BSA), serum creatinine concentration, serum cystatin C concentration, and absolute estimated GFR (AGFR) to the base model. GFR was estimated via the Chronic Kidney Disease Epidemiology Collaboration (CKD-EPI) 2012 equation [18] and adjusted to AGFR based on individual BSA, determined using the Mosteller formula [19].

Covariates that resulted in a significant decrease (*p* < 0.05, chi-squared distribution with one degree of freedom) of at least 3.84 in the OFV from the basic model and a reduction in the variability of the PK parameter were included. All significant covariates were simultaneously integrated into a comprehensive “full” model. Subsequently, each covariate was individually removed from the “full” model. If the increase in OFV exceeded 6.64 (*p* < 0.01, chi-squared distribution with one degree of freedom), indicating significant association with the PK parameter, it was retained in the final model.

### Model evaluation

Model validation was based on graphical and statistical criteria. Goodness-of-fit (GOF) plots, which included observed versus population prediction; observed versus individual prediction; conditional weighted residuals (CWRES) versus time and CWRES versus population prediction were initially used for diagnostic purposes [20]. The stability and predictive performance of the final model were further validated by non-parametric bootstrap analysis and visual predictive check (VPC) techniques [21, 22]. For the bootstrap analysis, resampling was repeated 1000 times, and medians and 95% confidence intervals (CIs) for the estimated parameters obtained from the bootstrap procedure were compared with the final model estimates [23]. Regarding the VPC, 1000 datasets were simulated using the final population model parameters, and 95% CIs for 2.5^th^, median (50^th^), and 97.5^th^ percentiles of simulated data were calculated and then compared with the observations [21, 22].

### Calculation of individual average CL_R_ and individual half-live (t_1/2_)

The reliability of model parameter estimates were further evaluated by comparing them to previous NCA results [3]. While most parameters could be directly compared, the comparison of CL_R_ between NCA and popPK analysis required special consideration due to its dependence on concentration and changes over time in cases of saturable/nonlinear elimination. In the popPK analysis, individual average CL_R_ values were derived from the empirical Bayes estimates (EBEs) generated by our final model. The EBEs were obtained by integrating the Michaelis-Menten-type nonlinear renal clearance over the observation time period and dividing by the duration of the observation period. Therefore, this provides the average CL_R_ values during the observation period for each subject, accounting for the plasma concentrations of adefovir as described by the model.

Furthermore, individual t_1/2_ of adefovir was computed using the formula: ${t}_{1/2}= \frac{0.693\times V}{CL}$, where CL represents individual total body clearance derived from CL_R_ + CL_NR_. Here, CL_R_ represents the aforementioned individual values, while CL_NR_ and the volume of distribution (V) represent the individual EBEs obtained from the final model.

## Results

Twenty-four healthy subjects (14 female) with the mean ± SD BMI of 24.5 ± 3.10 kg/m^2^ and age of 40.4 ± 16.0 years completed the clinical trial [3], resulting in 1101 adefovir plasma and urine concentrations for the popPK analysis. Detailed information on the demographic characteristics of subjects is provided in Supplemental Table 1s. A total of 9 (0.817%) samples with concentrations lower than the LLOQs were removed from the analysis. Supplemental Fig. 1s shows the plasma concentration and urine excretion profiles of adefovir over time.

### Population pharmacokinetic analysis

#### Adefovir base model

Plasma and urine samples were jointly analyzed using a one-compartment model, incorporating first-order absorption (including lag time) and combined renal and nonrenal elimination. This model adequately described adefovir concentrations in both plasma and urine and was selected as the base model. Implementation of nonlinear renal elimination decreased the OFV by 25.2 compared to linear renal elimination. IIV was found to be significant on the maximum rate (V_max_) of nonlinear renal elimination and V. Introduction of IOV for apparent bioavailability, apparent absorption rate constant (K_a_), lag time, and V improved the model significantly (OFV reduced by 400, 212, 61.6, 235 points, respectively). The residual unexplained variability of both plasma and urine data was best described by proportional error models.

#### Covariate model for effects of co-treatment

All potential PK parameters were separately assessed for reference and test periods. As a result, a significantly higher apparent bioavailability was identified in the test period (point estimate 73.6%) compared to the published value of 59.0% [24] which was inputted for the reference period (drop in OFV by 7.39). Additionally, a significant decrease in apparent K_a_ was observed during the test period (2.29 h^−1^) compared to the reference period (5.18 h^−1^), resulting in a decrease of OFV by 9.11. Introducing additional PK parameters for reference and test periods separately did not result in significant model improvement.

#### Final model with demographic and physiological covariates

Several demographic and clinical parameters (age, body weight, body height, sex, BMI, BSA, serum creatinine and cystatin C concentrations, and AGFR) were tested as potential covariates on PK parameters. After stepwise covariate model building, AGFR on V_max_ was the only statistically significant covariate retained in the model (drop in OFV of 12.2 points). The final covariate model on V_max_ is therefore represented by ${{\text{TVV}}}_{{\text{max}}}={\theta }_{{\text{Vmax}}}\times ({\text{AGFR}}/105)$, where TVV_max_ is the typical value of V_max_ and 105 mL/min is the median AGFR. Using a power relationship instead of a proportional relationship to describe the effect of AGFR did not improve the model. The key model development steps are summarized in Table 2.

**Table 2:** Summary of key model development steps

**Model**	**Description**	**OFV**
**Base model**		
1	One-compartment model with first-order absorption (including lag time) and combined Michaelis-Menten type nonlinear renal and linear nonrenal elimination	6401.44
**Covariate model for effects of co-treatment**		
2	Base model with separate estimates of apparent bioavailability for reference and test periods	6394.05
3	Model 2 with additional separate estimates for apparent *K*_a_ for reference and test periods	6384.89
**Final model with demographic and physiological covariates**		
4	Final model with AGFR as a covariate on *V*_max_	6372.69

*OFV* objective function value, *K*_*a*_ first-order absorption rate constant, *AGFR* absolute estimated glomerular filtration rate

### Model evaluation

**Fig. 1:**
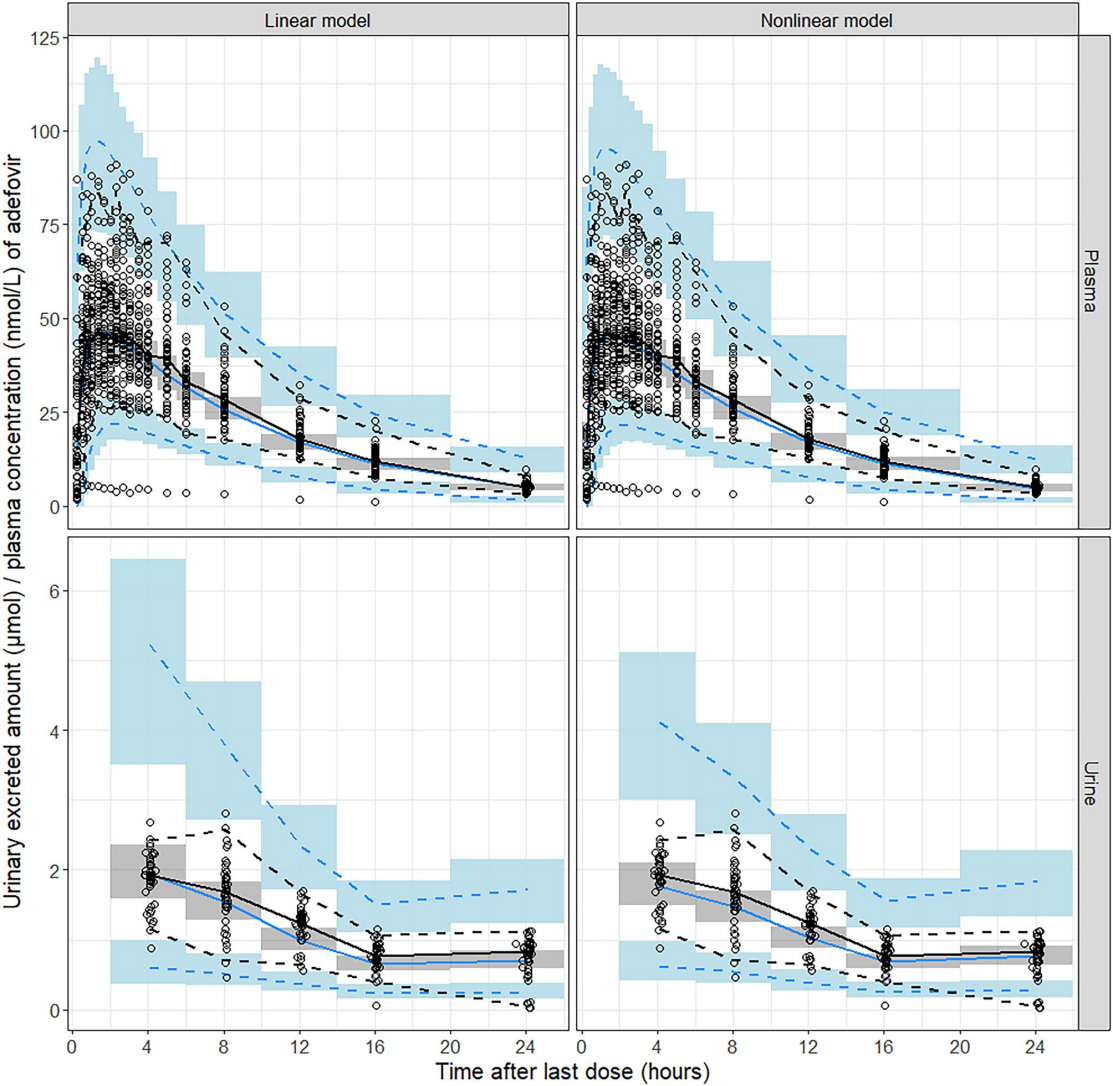
Visual predictive check (*n* = 1000) for the final model stratified by plasma and urine data, and categorized by renal elimination models: linear (“Linear model”) and nonlinear (“Nonlinear model”). Open circles illustrate observed data points. Solid (dashed) black and blue lines represent medians (2.5^th^ and 97.5^th^ percentiles) of observations and simulated data, respectively; blue, gray, and blue areas represent 95% confidence intervals of the 2.5^th^, median, and 97.5^th^ percentiles of simulated data

The VPC results (Fig. 1) showed that medians and 2.5^th^ and 97.5^th^ percentiles of the simulated data from the final model incorporating Michaelis-Menten-type nonlinear renal elimination (“Nonlinear model”) were in acceptable agreement with the observations. To facilitate a comparison between models with and without nonlinear renal elimination, an additional VPC is presented. This VPC is based on a linear renal elimination model (“Linear model”), which also incorporates AGFR as a covariate using a proportional equation, this time on CL_R_, to adjust for individual differences in renal function. Apart from a higher population variability in simulated compared to observed urine excretion amounts with both models, the VPC of the “Nonlinear model” shows no obvious misspecification, while it performs slightly better than the “Linear model,” particularly concerning the initial high adefovir amounts excreted in urine. The GOF plots (Supplemental Fig. 2s) demonstrated that the final model adequately described the observed adefovir plasma concentrations and urinary excretion, exhibiting a satisfactory fit without notable trends. This suggests the absence of systematic deviations in the model. The final point estimates and bootstrap statistics of PK parameters are summarized in Table 3. There are no indications of overparameterization in any of the model diagnostics or the bootstrap results.

**Table 3:** Population pharmacokinetic parameters of adefovir and bootstrap results

**Parameter (unit)**	**Point estimate**	**RSE %**	**Bootstrap median (95% CI)**
Fixed effects			
ALAG (h)	0.122	17.2	0.123 (0.0819–0.162)
*V* (L)	235	14.1	233 (196–321)
CL_NR_ (L/h)	11.8	25.7	11.6 (8.52–20.2)
*K*_m_ (nmol/L)	170	26.3	170 (122–295)
*V*_max_ (µmol/h)	2.40	23.5	2.42 (1.80–3.88)
Impact of co-treatment			
*F*_R_	0.590 fixed	–	–
*F*_T_	0.736	13.5	0.731 (0.629–0.999)
*K*_aR_ (h^−1^)	5.18	20.5	5.14 (3.54–7.74)
*K*_aT_ (h^−1^)	2.29	17.6	2.30 (1.66–3.30)
Inter-individual variability (CV%)			
*V*	17.1	22.1	16.3 (10.5–21.8)
*V*_max_	19.1	42.2	18.4 (6.32–29.6)
Inter-occasion variability (CV%)			
*F*	28.5	33.8	28.0 (11.6–48.1)
*K*_a_	104	14.9	101 (69.1–150)
ALAG	76.9	32.9	75.4 (43.0–129)
*V*	8.30	32.3	8.29 (4.90–11.2)
Residual unexplained variability (CV%)			
Proportional error, plasma	9.50	7.57	9.45 (8.23–10.9)
Proportional error, urine	28.1	18.7	27.7 (17.9–39.5)

*RSE%* relative standard error in percent, *CI* confidence interval, *ALAG* lag time for first-order absorption, *V* volume of distribution, *CL*_*NR*_ linear nonrenal clearance, *K*_*m*_ Michaelis-Menten constant for nonlinear renal elimination, *V*_*max*_ maximum rate of nonlinear renal elimination, *F* bioavailability, the bioavailability of the reference and test periods are represented by *F*_R_ and *F*_T_ respectively, *K*_*a*_ first-order absorption rate constant, the first-order absorption rate constant of the reference and test periods are represented by *K*_aR_ and *K*_aT_, *CV%* coefficient of variation in percent, *CV%* for inter-individual and inter-occasion variability computed as $\sqrt{exp\left({\omega }^{2}\right)-1}$, *CV%* for residual unexplained variability computed as $\sqrt{exp\left({\sigma }^{2}\right)-1}$

### Calculation of individual average CL_R_ and individual t_1/2_

The median (range) individual average CL_R_ and individual t_1/2_ values for adefovir during the reference and test periods were 12.4 (7.97–34.0) L/h and 6.59 (5.61–8.45) h, and 12.2 (7.82–31.0) L/h and 6.35 (5.07–9.01) h, respectively.

## Discussion

The present popPK analysis identified that changes in apparent absorption rate and apparent bioavailability, rather than changes in renal elimination, are the primary causes of the slight DDI that was found for adefovir as a victim when co-administered with other transporter probe substrates. Although the identified nonlinear renal clearance of adefovir is not saturated at the standard dose, further dose reduction in the existing transporter phenotyping cocktail might avoid even minor DDIs.

In this evaluation, plasma and urine data could be reasonably explained by a one-compartment model with first-order absorption (including lag time) and a combination of Michaelis-Menten-type nonlinear renal and linear nonrenal elimination. Despite previous studies reported that adefovir plasma levels declined biexponentially [10, 25, 26], our investigation did not reveal an observable biphasic decline in the semi-log plots (Supplemental Fig. 1s). Introducing a second compartment to the one-compartment base model did not significantly improve the BIC score (drop in BIC by 1.44), residual unexplained variability, or VPCs. Therefore, the one-compartment model was chosen as the final model.

The estimates for PK parameters of adefovir we present here are in line with the previous NCA conducted on the same dataset [3]. According to the final model, the individual apparent volume of distribution (V/F) for adefovir was calculated based on the individual EBEs of V divided by individual EBEs of F. The median of these individual values was 369 L and 308 L in the reference and test periods, respectively. These values are comparable to the geometric mean values obtained from the previous NCA study, which reported 368 L and 307 L for the corresponding periods [3]. Additionally, the median (range) CL_R_ and t_1/2_ values for adefovir during the reference and test periods in this evaluation are in accordance with those reported in the NCA study [3]. According to the NCA, during the reference period, the geometric mean (95% CI) CL_R_ and the geometric mean (95% CI) t_1/2_ of adefovir were 12.3 (6.06–24.9) L/h and 6.38 (4.68–8.72) h, respectively. In the test period, these values were 11.3 (7.26–17.8) L/h and 6.58 (5.05–8.57) h, respectively [3].

In our analysis, after normalizing for body weight, median individual values for V/F, CL_R_, and CL/F are 4.89 L/kg, 0.175 L/h/kg, and 0.493 L/h/kg, respectively. Moreover, the median urinary recovery over 24 h is 46.4%. These findings align with the characteristics reported in the summary of product characteristics for HEPSERA^®^ (adefovir dipivoxil) tablets [23] and are consistent with observations by Sokal et al. [26]. Sokal et al. studied adolescents aged 12–17 years, finding mean ± SD values of 0.739 ± 0.192 L/h/kg for CL/F, 7.16 ± 1.6 L/kg for V/F, and 6.84 ± 0.97 h for t_/2_ after a single oral dose of 10 mg adefovir dipivoxil [26]. However, our results were slightly lower than those reported by Hughes et al. [15] in infants and children (3 months–18 years) after a 1.5 mg/kg adefovir dipivoxil dose, showing a median CL/F of 1.0 L/h/kg and a median V/F of 8.1 L/kg. Conversely, our estimates of CL/F and V/F were slightly higher than those reported by Shiffman et al. [27] in patients with mild renal impairment (creatinine clearance ≥ 50 to < 80 mL/min), reflecting a mean CL/F of 0.270 L/h/kg and a mean V/F of 2.6 L/kg [27]. These discrepancies may stem from physiological variations due to factors such as growth, development, and disease.

In the study by Cundy et al. [10], a comparable mean ± SD value for CL_R_ was observed at 0.205 ± 0.078 L/h/kg. Nevertheless, they reported a lower V at 0.418 ± 0.076 L/kg, a shorter t_1/2_ of 1.6 ± 0.5 h, and a reduced CL of 0.223 ± 0.053 L/h/kg compared to our study. This discrepancy was noted following the intravenous administration of adefovir at 1 or 3 mg/kg/day in HIV-infected patients [10]. Considering adefovir dipivoxil, an ester prodrug of adefovir, may rapidly convert to adefovir after administration [28], it is not probable that adefovir dipivoxil pharmacokinetics play an important role in estimating the PK parameters of adefovir. Therefore, the underlying mechanisms of this difference remain unclear.

While Sun et al. [14] and Huang et al. [25] reported _1/2_ values similar to those in our study, Sun et al. presented notably low mean values for / (7.0 mL/kg) and CL/ (0.63 mL/h/kg) following a 10 mg oral dose of adefovir dipivoxil [14]. These values diverge from the mean serum concentration–time curves presented in their manuscript, prompting us to consider a potential unit mislabeling in the published results—suggesting “L” may be more appropriate than “mL.” Should this correction prove accurate, it would better align with the results of our current study. In contrast, Huang et al. reported a higher CL (1.00 L/h/kg) and a higher V (10.7 L/kg) after a 10 mg dose of adefovir dipivoxil [25]. We attribute this discrepancy to an error in their article, where they indicate, “open circles represent observed adefovir dipivoxil concentrations” [25]. It appears they inadvertently used adefovir dipivoxil as the moiety for concentrations instead of adefovir, inflating both and CL in their analysis. Overall, published data on adefovir pharmacokinetics show a remarkable variability in clearance and volume of distribution.

To evaluate the influence of co-treatment on PK parameters, we employed two approaches. Firstly, we independently evaluated PK parameters for reference and test periods. Additionally, we integrated co-treatment effect as a covariate on the PK parameters. The relative differences in PK parameters between these periods, as estimated by both methods, are consistent and resulted in an equivalent reduction of the OFV. To allow for greater flexibility in identifying potentially more fundamental distinctions in the description of PK processes between periods, we finally conducted a separate assessment of PK parameters for each of the reference and test periods, respectively. After a detailed assessment of the parameters in various PK processes using the popPK model, a higher apparent bioavailability, but a slower apparent absorption rate of adefovir when co-administered with the cocktail, has been identified. These findings indicate potential changes in the apparent absorption of adefovir dipivoxil or prodrug conversion, contributing to increased systemic exposure during co-administration with the cocktail, as observed in the previous DDI study [3]. Based on sporadic studies, a potential mechanism to explain this result may stem from the co-administered drugs exerting inhibitory effects on the intestinal multidrug resistance-associated protein 2 (MRP2, ABCC2). This protein mediates unidirectional transport of adefovir to the intestinal lumen within enterocytes [29–31]; thus, inhibition may enhance the bioavailability of adefovir. Furthermore, the model-based approach did not support the inclusion of V_max_ for reference and test periods separately. Additionally, no significant difference was observed in the median CL_R_ between reference and test periods. This suggests that CL_R_ remains unaffected by the other concomitantly administered probe drugs and may reliably reflect changes in the renal OAT1 activity, which is rate limiting for adefovir renal excretion [2, 9, 10].

The final model, incorporating a nonlinear renal elimination, provides an appropriate description of the data, as supported by the supplemental figures. The adefovir renal clearance plot (Supplemental Fig. 3s) indicates a deviation from linearity in urinary excretion at high concentrations, which is well described by the nonlinear renal clearance model. The residual plot (Supplemental Fig. 4s) illustrates clearly biased descriptions of urinary excretion with a linear model at high concentrations, supporting that the linear clearance model is inadequate. Furthermore a model with nonlinear renal elimination offers a physiologically plausible representation for adefovir renal elimination. Adefovir primarily undergoes OAT1-mediated tubular secretion, a process that could potentially saturate and complement kidney filtration. Calculated as CL_R_ – fu*GFR (with a median GFR of 6.28 L/h in this study) [2], adefovir secretion accounts for over 50% of CL_R_ in this study, aligning with findings in previous reports [10, 24]. The population estimate (95% CI) for K_m_ in nonlinear renal elimination is 170 (122–295) nmol/L, which is lower than in vitro studies (mean ± SD 23.8 ± 4.2 µmol/L) [11, 12]. This disparity may stem from challenges in replicating the dynamic in vivo environment in controlled in vitro settings and differences in techniques. Despite this, it exceeds the observed adefovir C_max_ range (5.56–91.0 nmol/L). Thus, this mechanism is expected to have little influence on systemic exposure and is considered clinically insignificant at therapeutic doses. However, this result suggests that a reduction of adefovir dose as part of the transporter probe cocktail may be considered in future studies to prevent relevant transporters to be saturated, and to minimize any impact on other cocktail components.

As discussed in the “Introduction” section, indeed there is some prior evidence for nonlinearity on adefovir pharmacokinetics [14, 15], albeit nonlinear renal clearance has not been shown yet [10, 25–27, 32–34]. The possible reason might be that such finding requires a popPK evaluation to gain a more detailed understanding of the role of individual PK processes for the pharmacokinetics of adefovir, while most of previous studies assessed the PK of adefovir by noncompartmental methods, which may provide insufficient information of individual PK processes [10, 14, 27]. Another possible reason could be that the extent of saturability in adefovir’s elimination, based on the recommended single dose of 10 mg, was relatively small. Therefore, both plasma measurements and urine collection might be necessary for a sound estimation of the nonlinearity. This could explain why Jihan Huang reported first-order elimination for adefovir in their popPK study [25].

The model could not be improved further by incorporating the AGFR as a component of CL_R_ in addition to nonlinear renal clearance. Although it might better reflect the physiological conditions of the renal excretion of adefovir, which involves both active tubular secretion and glomerular filtration, this approach resulted in unstable parameter estimates of K_m_ and V_max_ (RSEs > 1000%). As a result, we were unable to estimate the K_m_ value of true renal secretion, which might become more saturated at therapeutic adefovir concentrations.

Another limitation of this study is that it only included healthy adults who received a standard dose. To gain a comprehensive understanding of the nonlinear renal elimination of adefovir, future studies would need to include more dose levels, particularly higher doses, in diverse populations with varying degrees of renal function.

## Conclusion

In conclusion, the popPK modeling approach showed that the minor DDI observed for adefovir as a victim when co-administered with other transporter probe substrates is caused by an effect on apparent absorption and/or formation of adefovir from the prodrug, but not by an effect on renal elimination. Renal elimination of adefovir was found to be saturable, which should reflect its active renal secretion. The high K_m_ value suggests that the use of renal elimination of adefovir to assess renal OAT1 activity is not compromised by saturability, while using a lower adefovir dose (e.g., to 50%) may provide an additional safety margin.

### Supplementary Information

Below is the link to the electronic supplementary material.

Supplementary file1 (DOCX 8628 KB)

## Data Availability

The data that support the findings of this study are available from the corresponding author upon reasonable request.
