# Supplementary material for: Understanding adefovir pharmacokinetics as a component of a transporter phenotyping cocktail
Source: Eur J Clin Pharmacol. 2024 Mar 28;80(7):1069–78. doi: 10.1007/s00228-024-03673-x (PMC11156719; doi:10.1007/s00228-024-03673-x)
Supplement: Supplementary file 1 — Supplementary file1 (DOCX 8628 KB) [file 228_2024_3673_MOESM1_ESM.docx]

**Title:** Understanding adefovir pharmacokinetics as a component of a transporter phenotyping cocktail

**Journal:** European Journal of Clinical Pharmacology

**Author:** Qian Dong^1^, Chunli Chen^1, 2^, Max Taubert^1^, Muhammad Bilal^1,3^, Martina Kinzig^4^, Fritz Sörgel^4^, Oliver Scherf-Clavel^5^, Uwe Fuhr^1^, Charalambos Dokos^1^

**Institution:**

1. Faculty of Medicine and University Hospital Cologne, Center for Pharmacology, Department I of Pharmacology, University of Cologne, Cologne, Germany.

2. Heilongjiang Key Laboratory for Animal Disease Control and Pharmaceutical Development, College of Veterinary Medicine, Northeast Agricultural University, 600 Changjiang Road, Xiangfang District, Harbin 150030, PR China.

3. Department of Clinical Pharmacy, Institute of Pharmacy, University of Bonn, Bonn, Germany.

4. Institute for Biomedical and Pharmaceutical Research, Nürnberg-Heroldsberg, Germany.

5. Ludwig-Maximilians-Universität München, Department Pharmazie, Butenandtstr. 5, 81377 München, Germany.

**Corresponding author:**

Qian Dong

Department I of Pharmacology, Center for Pharmacology, Faculty of Medicine and University Hospital Cologne, University of Cologne, Gleueler Straße 24, Cologne 50931, Germany

Email: qdong2@smail.uni-koeln.de

Phone: +49-(0)-221-478-6672

**Keywords:** adefovir, population pharmacokinetics, nonlinear renal elimination, OAT1-mediated drug-drug interactions

**SUPPLEMENT**

**Supplemental Table 1s** Demographic characteristics of subjects

| **Demographic characteristics** | **No. or median (range)** |
| --- | --- |
| No. of subjects (male/female) | 24 (10/14) |
| Age (y) | 35 (20 - 68) |
| Body weight (kg) | 71.3 (55.5 - 94.9) |
| Body height (cm) | 172 (160 - 188) |
| BMI (kg/m^2^) | 24.5 (19.5 - 29.7) |
| BSA (m^2^) | 1.83 (1.59 - 2.18) |
| AGFR (mL/min) | 105 (77.5 - 136) |
| Serum creatinine concentration (mg/dL) | 0.855 (0.660 - 1.14) |
| Serum cystatin C concentration (mg/L) | 0.835 (0.660 - 1.10) |

*BMI* body mass index, *BSA* body surface area, determined using the Mosteller formula [1], *AGFR* absolute estimated glomerular rate. Glomerular filtration rate (GFR) was estimated using the Chronic Kidney Disease Epidemiology Collaboration (CKD-EPI) 2012 equation [2] and adjusted to AGFR based on individual BSA [1].


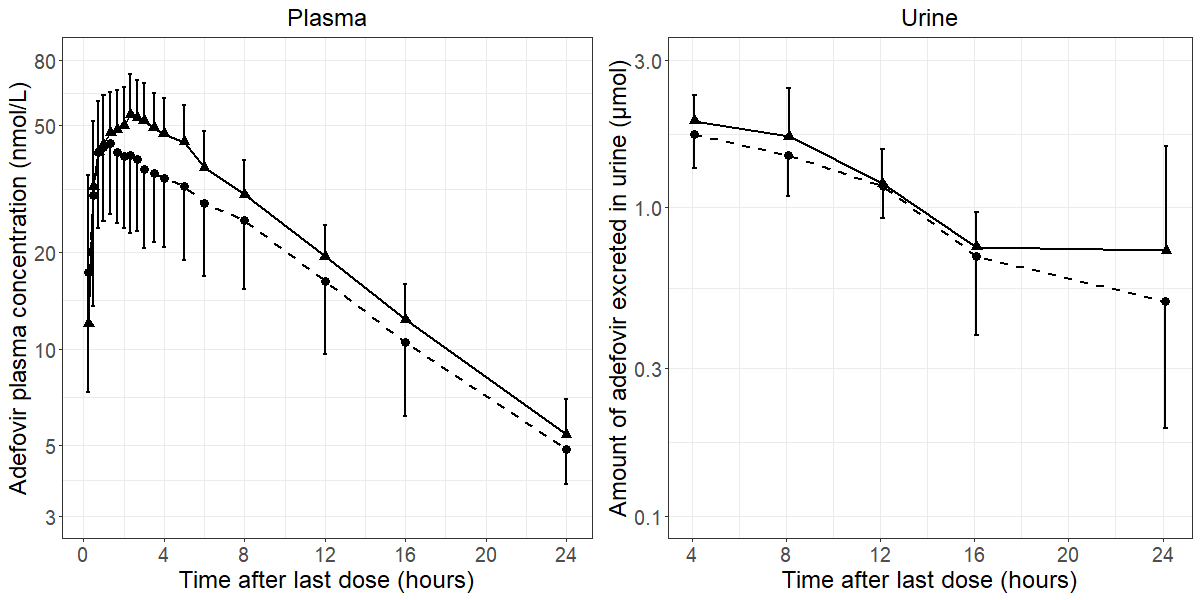


**Supplemental Fig. 1s** Semi-logarithmic plots illustrate the adefovir plasma concentration and the amount of adefovir excreted during the urine collection period over time in both the reference (circle points and dashed lines) and test (triangle points and solid lines) periods. The associated symbols and error bars represent geometric means and geometric standard deviations (SD), respectively


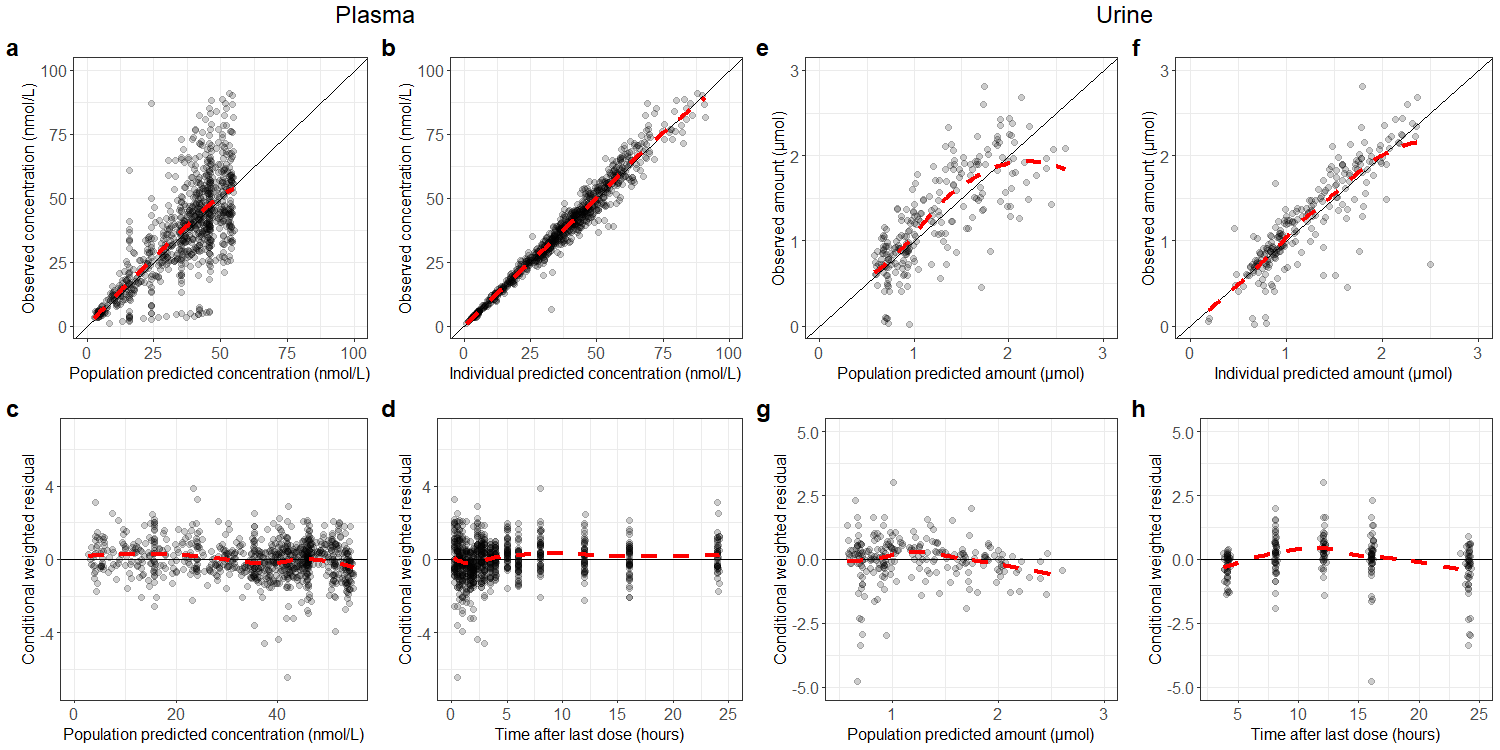


**Supplemental Fig. 2s** Goodness-of-fit plots of the final model for plasma concentrations (a-d) and amounts excreted in urine (e-h). a, e: Population predicted *versus* observed for plasma concentrations and amounts excreted in urine; b, f: Individual predicted *versus* observed for plasma concentrations and amounts excreted in urine; c, g: Conditional weighted residual *versus* population predicted for plasma concentrations and amounts excreted in urine; d, h: Conditional weighted residual *versus* time after last dose for plasma concentrations and amounts excreted in urine. Red dashed lines represent locally weighted smoothing lines.


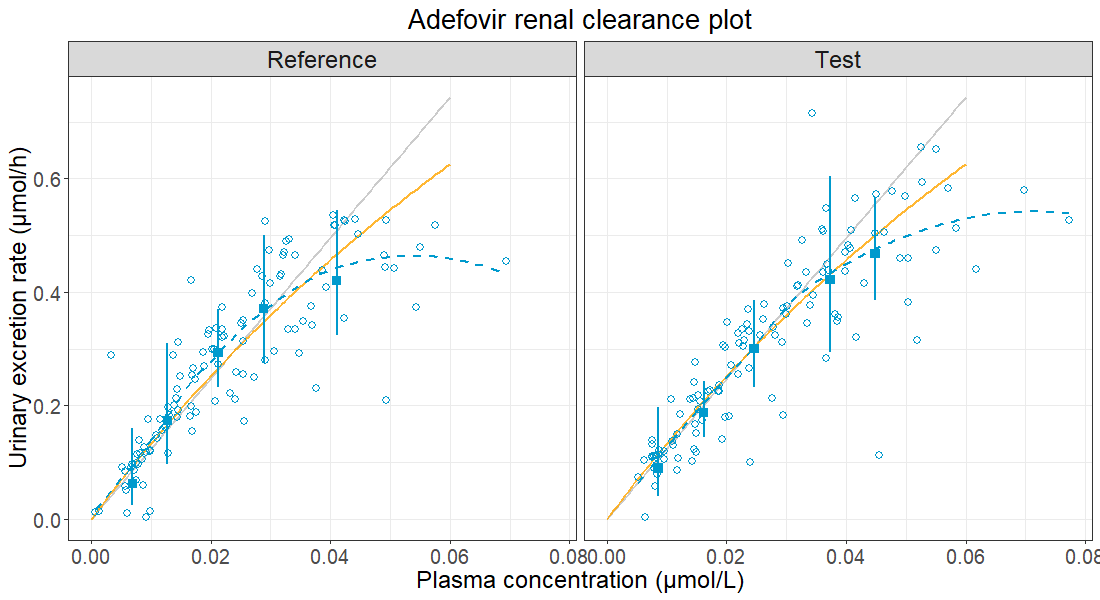


**Supplemental Fig. 3s** Adefovir renal clearance plots illustrate a comparison between observed and estimated urinary excretion rates using both nonlinear and linear renal elimination models against the geometric mean of individual average adefovir plasma concentrations across the five urine collection intervals in both reference and test periods. The blue open circles represent individual observations of urinary excretion rate *versus* average plasma concentrations in each urine collection interval. The individual average plasma concentrations result from dividing the area under the curve of observed plasma concentrations by the duration of each urine collection interval. Dashed blue lines represent locally weighted smoothing lines of these individual observation pairs. Blue solid square points and error bars depict geometric means and ± one geometric SD of observed individual urinary excretion rates in comparison to the geometric means of individual average plasma concentrations during each of the five urine collection intervals. Orange and grey lines represent population predictions of renal excretion rate from the nonlinear and linear models, respectively.


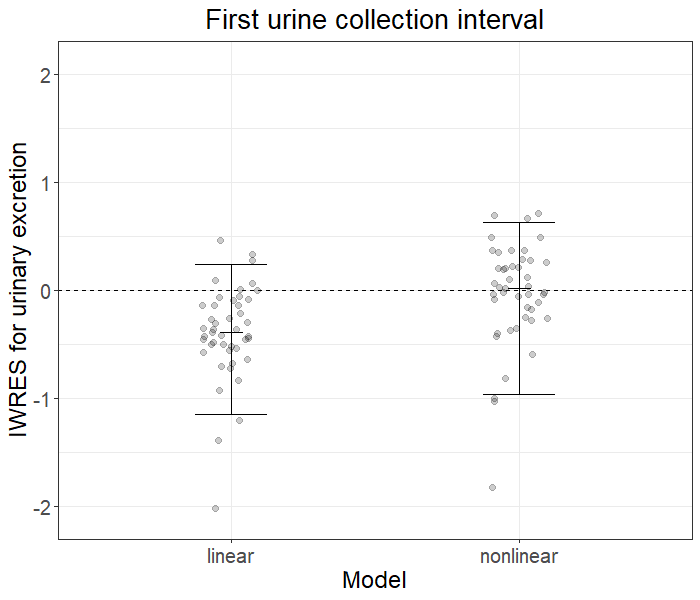


**Supplemental Fig. 4s** Residual plots compare the urinary adefovir excretion for both the linear and nonlinear models during the first urine collection interval. Scatter points represent individual weighted residuals (IWRES) calculated from the final nonlinear and linear models. Error bars show the 5^th^, 50^th^, and 95^th^ percentiles of the data.

**References**

1 Mosteller R (1987) Simplified calculation of body surface area. New Engl J Med 317: 1098 DOI 10.1056/NEJM198710223171717

2 Inker LA, Schmid CH, Tighiouart H, Eckfeldt JH, Feldman HI, Greene T, Kusek JW, Manzi J, Van Lente F, Zhang YL (2012) Estimating glomerular filtration rate from serum creatinine and cystatin C. New England Journal of Medicine 367 (1): 20-29 DOI 10.1056/NEJMoa1114248
